# Research on Rheumatic Diseases

**Published:** 1961-01

**Authors:** G. D. Kersley


### RESEARCH ON RHEUMATIC DISEASES

OR

### "WHAT'S COOKIN' AT BATH?"

BY

G. D. KERSLEY, T.D., D.L., M.D., F.R.C.P.

It was within the Royal National Hospital for Rheumatic Diseases that the research unit, whose activities are to be reviewed here, began its existence in 1949. But the hospital had been famous, and had been engaged in research, for many years before that. Before the last war, it was the Mecca of students of rheumatic disesase from all over the world. It is the oldest spa hospital in the world, and was the first to specialize entirely in rheumatic diseases.

The Hospital's history begins with the opening of the subscription list by Beau Nash in 1723. Ralph Allen provided the stone, and John Wood the Elder gave his services as architect, free. In 1742 the hospital was opened for 150 patients from outside the City of Bath, under the name of "The Hospital or Infirmary at Bath", with Dr. Oliver (of Bath Oliver Biscuit fame) as the first physician and Mr. Pierce as the first surgeon. After this, among the subscriptions received one of special interest was of £1,000 from the Guildhall, London, for services to those wounded in the 'Forty-Five rebellion, and one of £29,280 from a Dr. Perry. First, an extra floor containing a pool was built and then in 1861 a complete new wing was added. Before this the patients went to the City "baths", if necessary by sedan chair, but they wore a token of brass to show that they were patients of the Hospital and were not to be served with beer *en route*. In 1887 the Hospital received its Royal Charter from Queen Victoria, and in 1911 His Majesty King Edward VII became Patron. In 1935 it was decided to build a new hospital and His Majesty approved the change of name to the Royal National Hospital for Rheumatic Diseases. In 1939 Her Royal Highness the Duchess of Kent, then our President and now our Patron, visited the Hospital and accepted purses as contributions to the rebuilding fund. In 1942 the west wing received a direct hit in an air raid and was partly destroyed. Under the National Health Service Act of 1946 the funds collected for rebuilding were appropriated into the common pool. Since then every effort has been made, first to obtain permission to build a new hospital as previously planned on the prepared site, and then, when the permission was withheld, to obtain sanction for the proper reconstruction of the old building. Firstly a deputation appealed to the Minister of Health, then a Press Conference in London received phenomenal support, especially from *The Times*, and finally the efforts of an all-party deputation of M.P.s were crowned with success. It is only this year, after 25 years of planning and frustration that the Ministry of Health has sanctioned the "reconditioning" of the hospital, with an extra half-floor, at the expenditure of approximately a quarter of a million pounds. Detailed plans and quantities are now being drawn up and it is hoped to commence the alterations next spring and complete them in about three years' time. After rebuilding, with the preservation of the eighteenth century façade and hall, the hospital will have only a hundred beds, but will then have all "mods. cons." screened beds, properly grouped treatment departments and better intercommunications with proper bed lifts. A new third floor will house a minor operating theatre and a plaster room and we hope new clinical research facilities. It will cease to be the pre-Victorian death-trap in which we have worked for so many years. This hospital has proved how little bricks and mortar really count. Lord Horder in 1938 stated that it was doing better work in rheumatism than any other in the world, though the building was fit only for rats to live in!

But to return to our subject of research—this of course started long before the National Health Act and the foundation of the Unit. Apart from early contributions on gout, the Royal National Hospital in 1930 led the way in the use of plaster techniques in the splinting of joints and in 1935 a Governor, Mr. Sidney Robinson, paid for a whole-time research worker, Dr. Levinthal, to carry out fundamental work on the immunological conception of rheumatoid arthritis—work which fits in well with some of our newest concepts.

When the Rheumatism Research Unit was founded in 1949 it was designated as belonging to the South West and to the Oxford Regions, though it was always financed by the South West Regional Hospital Board. It grew in perhaps a somewhat untidy fashion financially, but it had a very happy and productive childhood. Clinical work, clinical research, and fundamental research were so closely bound together as to be, at times, almost indistinguishable. Clinical registrars to the unit helped with research, and research fellows of the same status helped with clinical work. As all aspects were financed by the Regional Board with the help of external grants obtained from the Nuffield Foundation, Empire Rheumatism Council, and personal gifts and legacies, everyone was happy. Even though part of the work was carried out in the unused hospital mortuary, keenness and co-operation overcame these difficulties. In 1951 however part of the unit moved to new quarters on the Combe Park Site.

From 1st April 1959, the fundamental research has been taken over by the Medical Research Council with a local Advisory Committee of Directors, consisting of four Professors from Bristol University, three Bath physicians and a member of the Medical Research Council. This has of course given the unit a new status, but it has dropped an "iron curtain" between fundamental and clinical research in all matters of finance. It is however hoped that the barrier will exist only at the level of finance, as all research workers agree that both the scientist and the clinician gain enormously by working in the closest possible physical and psychological contact.

It is difficult to make a readable review of fundamental research and its intricate facts and theories, which are "Greek" except to those recently trained in biochemistry and its methodology. From early beginnings on fibrinolysis and tryptolytic enzymes, the Unit concentrated more on methods of tissue culture and on the *in vitro* changes occurring during tissue growth, its accelerators and inhibitors. Normal and rheumatoid synovial membrane was examined in this way, and various techniques for studying artificially produced arthritis in animals were investigated. Animal work with rats, guinea pigs, and rabbits was carried out on the utilization, excretion, and deposition of gold, using radio-active isotopes, in order to add to knowledge of auro-therapy [8, 16].

The early post-war clinical research carried out at the Royal National Hospital was concerned with the histology of rheumatoid nodules, changes in skeletal muscles, and degenerative changes in tendons leading to their rupture [1, 9, 10, 12]. This was followed by work on dental sepsis in rheumatic disease [11], the use of blood transfusion in rheumatoid arthritis, [21] and a considerable number of observations on pituitary stimulation with insulin hypoglycaemia or E.T.C. and its biochemical consequences [13, 14]. An experiment with a well-known vaccine showed that the psychological effect of the regular injection of the saline control was of no mean value [15]. The anarthritic syndrome of the elderly [16] and an episodic rheumatoid onset often confused with palindromic rheumatism and intermittent hydrarthrosis [1] were described.

Much clinical research consists of clinical trials, in the planning of which a definite discipline has to be followed. The purpose of the trial and the type of case to be admitted must be defined. In some trials where quick results are expected, the patient may act as his own control and the value of different remedies may be compared by a "blind" change in treatment. As a rule, however, control groups selected by random sampling are essential, and neither the patient nor the assessor should know the drug the patient is taking. Every effort must be made to neutralize possible psychological factors. Statistical analysis of the data is essential. The assessor should be the same for each recording—preferably a research fellow of senior registrar grade—but the over-all welfare of the patients must remain the responsibility of a physician who, on ethical and humanitarian grounds, must be prepared to put the patient before the project and withdraw him from the trial, noting the reason for withdrawal, if he thinks it is to the patient's advantage.

The first major clinical trial was concerned with the effects of radiotherapy [2]. Rheumatic patients in a carefully controlled series were given various dosages of X-ray therapy, and in the control group the patients were laid on a couch without the current being turned on. It was found that those with rheumatoid arthritis received no benefit, about 50 per cent of osteo-arthritics had some temporary amelioration of symptoms, and that 95 per cent of those with ankylosing spondylitis were greatly improved, the benefit lasting in the majority of patients for at least one year. The medium range of dosage, about 1,200 roentgen units, was found the most suitable. This investigation was carried out before the leukaemia dangers of radio-therapy had been discovered, but now the knowledge that radiography increases the chance of developing leukaemia about seventeen-fold must be considered before it is used, especially as it is so frequently required in spondylitics in the comparatively young age groups. However, this treatment still appears justifiable where severe pain cannot be relieved by other methods.

During this same period a second clinical trial was carried out on the use of intra-articular injections in osteo-arthritis. It was found that some slight benefit in the way of increased mobility and reduction of pain was produced to an extent equal to that produced by "novocaine", saline or lactic acid. The control group suffered only needling of the joint.

A considerable amount of work [4, 5, 6, 7] was carried out on the anaemia usually associated with rheumatoid arthritis. It seemed that there were many factors involved—neither deficient absorption, blood loss, changes in iron binding, nor haemodilution could explain the anaemia. Parenteral iron improved a large proportion of patients resistant to iron by mouth. Cobalt assisted in a few resistant cases but gastric complications were frequent. Bone marrow findings were equivocal. It seems probable that deficient iron absorption, excessive iron requirements, and some haemopoietic abnormality all played their part in the anaemia of rheumatoid arthritis. To this may be added the small repeated blood losses due to aspirin treatment, probably insignificant except when long continued in the menstruating woman, whose safety margin is reduced.

Projects still in progress consist of investigation of the uricosuric treatment of gout, an evaluation of antimalarial drugs in rheumatoid arthritis, further work on the treatment of anaemia with iron, and the use of long-acting steroid injections, not yet on the market, in order to determine their dosage, length of action, and likely value when there is the complication of peptic ulceration.

In the antimalarial trial a series of patients suffering from rheumatoid arthritis was given plaquenil, camoquin, and control tablets for alternating periods [1]. A high percentage of patients enjoyed suppression of symptoms with the antimalarials, but the margin between toxicity and therapeutic benefit was very small, and one patient developed agranulocytosis as a result of camoquin treatment. Temporary punctate corneal opacities were common. It was felt that this group of drugs might be used with justification in a limited number of cases where safer, simpler measures are ineffective, provided careful supervision is maintained.

For some three years now a series of fifty patients with gout has been followed up clinically and biochemically while under treatment with a number of drugs which increase uric acid excretion [19, 20]. These consist of aspirin in large doses, probenacid

("benemid"), "longacid" (or "urelim"), "anturan" (G.28.315) and zoxazolamine ("flexin"). Aspirin is cheap, but in large doses it is depressing and is a gastric irritant; "benemid" is satisfactory in about 80 per cent of cases but produces toxic symptoms in the remainder. "Longacid" is very similar in its effect. "Anturan" is more powerful and virtually non-toxic. Zoxazolamine appears to run "anturan" a fairly close second as the uricosuric agent of choice. Apart from aspirin, all these uricosuric agents tend to stir up gouty attacks during the first six weeks of administration, but from thence onwards the attacks become less severe and less frequent, and any interval swelling is reduced. During these first six weeks therefore colchicine should be administered regularly, and butazolidin may be given in addition for two days at a time to control any severe attacks. Aspirin inhibits the effects of the other drugs, but the remainder are complementary to each other. Long-term and regular administration of uricosuric agents is essential to their effectiveness. They appear to have no significant effect on plasma cholesterol levels or prothrombin times, a possible drawback that has been suggested by some cardiologists. Their use has revolutionized the treatment and prognosis of severe gout.

Apart from research work carried out on its own initiative, the Unit has been participating for the past five years in various multi-centre trials organized by the Medical Research Council, Empire Rheumatism Council, and Nuffield Foundation. Some five to ten centres in the country may work on the same project, using the same criteria laid down by a central committee, and the results are assessed by one medical statistician. The first trial showed that in the long run little more benefit was to be expected from cortisone than from aspirin[22]. Prednisolone however produced considerable long-term benefit including less deterioration as seen on consecutive X-rays [23]. Gold therapy produce definite amelioration at the end of a year's follow-up, when the effects of a course of 1 gm. was compared with that of 0·01 mgm. [24]. These trials are still being continued to assess long-term results and toxic reactions.

As we said before, with the change in ownership of the unit a temporary iron curtain was dropped between the fundamental and clinical research. A chink in this curtain is beginning to appear in that clinical research facilities may be available in the rebuilt hospital, and the Medical Research Council may help with some of these, thus bringing such projects under the aegis of the Committee of Directors of the Unit. Likewise one day the unit's fundamental work may move back to its original home at the Royal National Hospital, so that once again it may stimulate the clinicians, and the latter in their turn may help the unit to keep its feet firmly planted on the ground, for in spite of recent theories on parthenogenesis, intercourse between the research worker and the clinician is still essential for productivity.

### REFERENCES

[1] Desmarais, M. H. L., Gibson, H. J. and Kersley, G. D. (1948). *Ann. rheum.* Dis., **7**, 132.
[2] Desmarais, M. H. L. (1953). *Ibid.*, **12**, 25.
[3] Desmarais, M. H. L. (1952). *Ibid.*, **11**, 277.
[4] Jeffery, M. R. (1952). *Ibid.*, **11**, 162.
[5] Jeffrey, M. R. (1953). *Blood*, **8**, 502.
[6] Jeffrey, M. R. (1955). *Clin. Sci.*, **14**, 395.
[7] Jeffrey, R. M. (1956). *Ann. rheum. Dis.*, **15**, 151.
[8] Jeffrey, R. M., Freundlich, H. F. and Bailey, D. M. (1955) *Ibid.*, **14**, 353.
[9] Kers.ey, G. D., Gibson, H. J. and Desmarais, M. H. L. (1946). *Ibid.*, **5**, 41.
[10] Kersley, G. D. (1948). *Brit. Med. J.*, *ii*, 942.
[11] Kersley, G. D. (1949). *Proc. Roy. Soc. Med.*, **42**, 151.
[12] Kersley, G. D., Barber, H. S., Cregan, J. C. and Gibson, H. J. (1954). *J. Bone Jt. Surg.*, **36**, 238.
[13] Kersley, G. D., Mandel, L., Jeffrey, M. R., Desmarais, M. H. L. and Bene, E. (1950). *Brit. Med. J.*, *ii*, 855.
[14] Kersley, G. D., Mandel, L., Jeffrey, M. R., Bene, E. and Taylor, K. B. (1951). *Brit. Med. J. ii*, 574.
[15] Kersley, G. D. (1956). *Contemporary Rheumatology*, European Rheumatology Congress 1955.
[16] Kersley, G. D. (1951). International Congress on Rheumatic Diseases, Barcelona: Report.
[17] Kersley, G. D. (1954). *Lancet*, *i*, 1206.
[18] Kersley, G. D. (1959). *Lancet*, *ii*, 886.
[19] Kersley, G. D., Cook, E. R. and Tovey, D. C. J. (1958). *Ann. rheum. Dis.*, **17**, 326.
[20] Kersley G. D. and Gibbs, A. R. (1960). *Ann. rheum. Dis.*, (in press).
[21] Simpson, N. W. R., Kersley, G. D. and Hall Brooks, B. (1949). *Ann. rheum. Dis.*, **8**, 277.
[22] Report (1955). *Ann. rheum. Dis.*, **14**, 353.
[23] *Ibid* (1960). **18**, 173.
[24] *Ibid* (1960). **19**, 95.

